# Aptamer‐Based DNA Allosteric Switch for Regulation of Protein Activity

**DOI:** 10.1002/advs.202402531

**Published:** 2024-06-12

**Authors:** Hongzhi Sun, Di Zhao, Yating He, Hong‐Min Meng, Zhaohui Li

**Affiliations:** ^1^ College of Chemistry Institute of Analytical Chemistry for Life Science Zhengzhou University Zhengzhou 450001 China; ^2^ The First Affiliated Hospital of Zhengzhou University Zhengzhou 450052 China

**Keywords:** allostery, aptamer, bionics, enzyme activity regulation, molecular switch

## Abstract

Allostery is a fundamental way to regulate the function of biomolecules playing crucial roles in cell metabolism and proliferation and is deemed the second secret of life. Given the limited understanding of the structure of natural allosteric molecules, the development of artificial allosteric molecules brings a huge opportunity to transform the allosteric mechanism into practical applications. In this study, the concept of bionics is introduced into the design of artificial allosteric molecules and an allosteric DNA switch with an activity site and an allosteric site based on two aptamers for selective inhibition of thrombin activity. Compared with the single aptamer, the allosteric switch possesses a significantly enhanced inhibition ability, which can be precisely regulated by converting the switch states. Moreover, the dynamic allosteric switch is further subjected to the control of the DNA threshold circuit for realizing automatic concentration determination and activity inhibition of thrombin. These compelling results confirm that this allosteric switch equipped with self‐sensing and information‐processing modules puts a new slant on the research of allosteric mechanisms and further application of allosteric tactics in chemical and biomedical fields.

## Graphical abstract

An artificial allosteric switch based on aptamer is fabricated through simulated allostery and the inhibition of thrombin by it is demonstrated as a case study. The allosteric switch possesses a significantly enhanced and adjustable inhibition ability. Moreover, the allosteric switch is plugged into the DNA circuit to achieve self‐sensing and selective inhibition of thrombin.

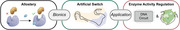

## 1 Introduction

Allostery, a phenomenon in the conformation and function of a biomolecule changes due to the binding of an effector at the allosteric site (that is, a site other than the active site), was deemed the second secret of life by Monod.^[^ ^1^, ^2^ ^]^ The allosteric effect is crucial to cellular metabolism, cell signaling, transcription and expression of genetic information, and other physiological processes by regulating enzyme activity.^[^ ^3^ ^]^ Therefore, subjecting the allosteric molecule to artificial regulation is of great significance to understanding the mechanism of allostery and developing allosteric modulators for disease treatment. Currently, researchers have zeroed in on experiment‐based characterization and analysis, as well as artificial intelligence‐assisted theoretical computation and structural prediction to explore potential druggable allosteric sites.^[^ ^4^, ^5^, ^6^ ^]^ However, few satisfactory results are obtained, and the discovery of allosteric sites is still full of challenges.^[^ ^4^ ^]^ A more promising road to understanding the allostery mechanism and realizing practical application is developing synthetic allosteric molecules since it does not share the limitations of their natural counterpart.

The three key components of a natural allosteric molecule are an allosteric site for identifying and binding effectors, an active site with variable conformation and function, and a connector that is enable to transmit signals between two sites. Hence, the performance and utilization potentiality of a synthetic allosteric molecule largely depend on the selected building frame and material that satisfy the above prerequisites. Nucleic acid, especially deoxyribonucleic acid (DNA), features complementary base pairing and programmability that has been favored considerably by researchers to build nanorobots,^[^ ^7^, ^8^ ^]^ logic circuit networks,^[^ ^9^, ^10^, ^11^ ^]^ and theranostic nanoplatforms.^[^ ^12^ ^]^ Some dynamic DNA structures based on strand displacement reaction or the assembly/disassembly of certain special modules like i‐motif and G‐quadruplex can implement conformation change in response to the external environment,^[^ ^13^, ^14^, ^15^ ^]^ and these changes could be transmitted by the switching between flexible single‐stranded DNA (ssDNA) and rigid double‐stranded DNA (dsDNA) for subsequent reconfiguration of the whole structure.^[^ ^16^, ^17^ ^]^ Therefore, it seems feasible to engineer synthetic allosteric molecules availing DNA nanomaterial for the study and further regulation of physiological function. Furthermore, researchers have demonstrated that the combination of the DNA circuit and dynamic DNA structure is a straightforward strategy for the development of intelligent DNA devices.^[^ ^18^, ^19^, ^20^, ^21^, ^22^ ^]^ Recently, the allosteric signal transduction modules constructed by Zhang et al. have manifested great potential in simulating natural signal pathways.^[^ ^23^, ^24^ ^]^ However, few studies have been done to intelligently manipulate synthetic allosteric molecules mediated by DNA circuits for spontaneous regulation of physiological processes.

**Scheme 1:**
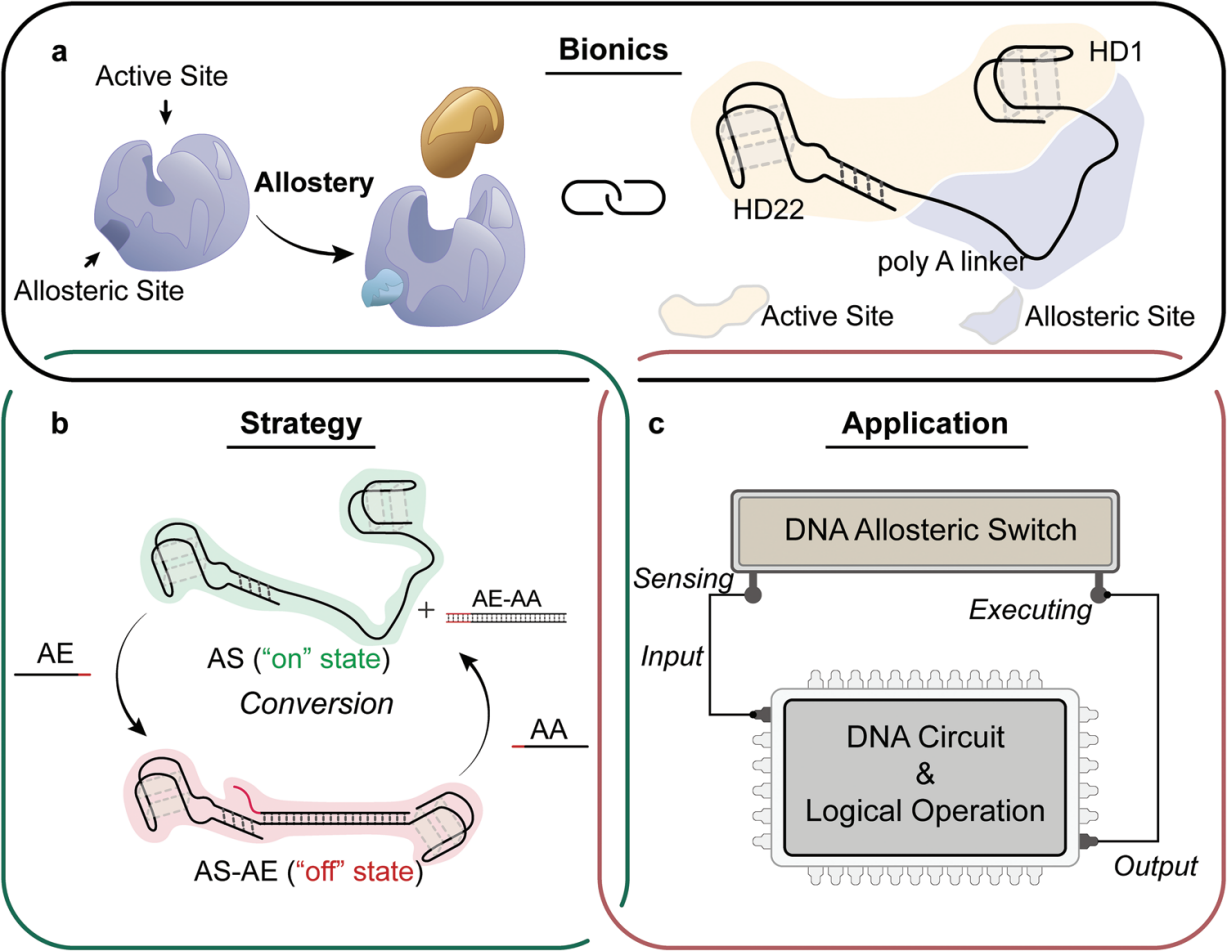
Schematic illustration of the designed aptamers‐based allosteric switch. a) The bionic allosteric switch, in which poly A linker is the allosteric site, and the entirety of two aptamers is the active site. b) The conformation and function conversion of the allosteric switch is controlled by the TMSD reaction due to the addition of the allosteric effector (AA) and allosteric antidote (AE). c) The allosteric switch is plugged into the DNA threshold circuit for the logic operation to realize the selective inhibition of thrombin activity.

Herein, we rationally designed a biomimetic allosteric switch with the aptamers as the fundamental elements, and selective inhibition of thrombin activity was demonstrated as a case study. As shown in **Scheme** **1**a, the allosteric switch (AS) consists of three segments of DNA strands: HD22, HD1, and a poly A linker. Among them, the poly A linker is the allosteric site counterpart of the natural allosteric molecules, and HD1 and HD22 are well‐studied aptamers that form the active site together. And it is worth mentioning that HD1 exhibits excellent inhibition of thrombin activity,^[^ ^25^ ^]^ while HD22 has a weak inhibition ability, but a more striking affinity to thrombin.^[^ ^26^ ^]^ The ssDNA and dsDNA can be converted into each other by the toehold‐mediated strand displacement (TMSD) reaction, accompanied by the change of the distance and orientation of the modified functional groups, which shares an identical mechanism with the allosteric effect. Therefore, an allosteric effector strand (AE) and an allosteric antidote strand (AA) were introduced to complete the conformational conversion of the allosteric switch (Scheme 1b). Whereafter, the allosteric switch was plugged into the DNA threshold circuit to achieve self‐sensing and selective inhibition of thrombin (Scheme 1c). Such an allosteric switch with convertible conformation and function gives new insights into the study of allosteric mechanisms in nature and the development of advanced artificial allosteric function units.

## 2 Results and Discussion

### 2.1 The Design of the Allosteric Switch

**Figure 1:**
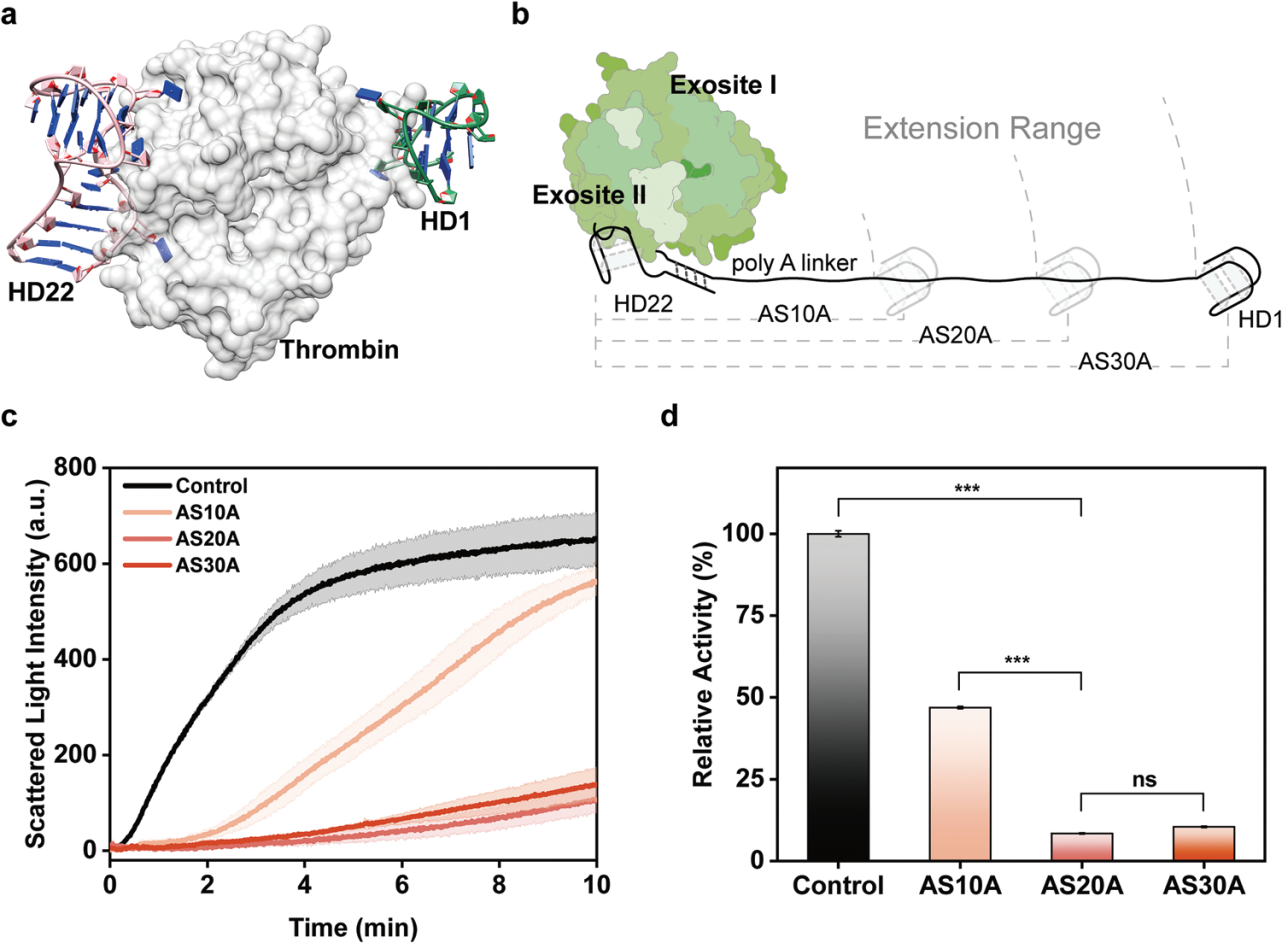
Optimization of poly A linker length in the synthetic allosteric switch. a) The spatial position of HD1 and HD22 binding to thrombin, respectively. HD1 binds to the fibrinogen‐recognition exosite (exosite I), while HD22 binds to the heparin‐binding exosite (exosite II). b) The linker length of the allosteric switch affects the binding of HD1 and HD22 to thrombin. The longer linker causes the allosteric switch to extend further, while the suitable linker endows the allosteric switch with a more powerful inhibition effect of thrombin activity. c) The kinetics of light scattering due to the conversion of fibrinogen (1.25 mg mL^−1^) into fibrin catalyzed by thrombin (0.75 U mL^−1^, Control) or thrombin that were preincubated with different allosteric switches (10 nm, ASnA, n = 10, 20, and 30). The fill area under the solid lines is the error bar (*n* = 3, mean ± SD). d) The relative activity of thrombin that was preincubated with different allosteric switches (*n* = 3, mean ± SD). ns, *p* > 0.05; ^***^ *p* < 0.001.

HD1 inhibits the coagulation potential of thrombin by binding the fibrinogen‐recognition exosite (exosite I), while HD22 binds to the heparin‐binding exosite (exosite II) with a strong affinity to thrombin.^[^ ^27^, ^28^, ^29^ ^]^ The inhibition ability of AS depends on the spatial conformation of two aptamers when they bind to thrombin. Therefore, the length of the linker was investigated first to ensure the successful construction of the allosteric switch. **Figure** **1**a shows the x‐ray structure of the complex between human alpha thrombin, HD1, and truncated HD22 (27‐mer).^[^ ^30^, ^31^, ^32^, ^33^ ^]^ The linear distance between the two binding sites is ≈4.4 nm (Figure S1, Supporting Information). To achieve the optimal inhibition effect, three poly A linkers with different lengths (10‐mer, 20‐mer, and 30‐mer) were used to constitute allosteric switches that were named AS10A, AS20A, and AS30A, respectively (Figure 1b).

To quantify the inhibition ability of ASnA (n = 10, 20, or 30), the kinetics of light scattering in 650 nm was measured, whose enhancement is caused by thrombin‐catalyzed fibrinogen coagulation (Figure 1c). AS20A and AS30A exhibited more significant anticoagulation than AS10A, indicating that the long enough poly A linker is crucial for simultaneous binding between thrombin and two aptamers. Furthermore, the relative activity is defined as the ratio of the initial rate of catalytic reaction between the ASnA‐incubated group and that of thrombin alone (control group), and the inhibition rate is defined as the ratio of the reduction between the two rates to the initial reaction rate of the control group (Equations (1) and (2)):

(1) $$Relative\;Activity\;=\frac{V_{0}\left(\mathrm{ASnA}\right)}{V_{0}\left(\mathrm{Control}\right)}\;\times 100\%$$

(2) $$Inhibition\;Rate\;=\frac{V_{0}\left(\mathrm{Control}\right)-V_{0}\left(\mathrm{ASnA}\right)}{V_{0}\left(\mathrm{Control}\right)}\;\times 100\%$$

The performance of allosteric switches is shown in Figure 1d. Despite AS30A having a comparable inhibition ability to AS20A, a lengthy linker may increase the cost of synthesis and the probability of encountering multiple thrombin molecules in solution leading to *two‐*to*‐two* or *n‐*to*‐n* combinations (Figure S2, Supporting Information).^[^ ^34^ ^]^ Henceforth, the allosteric switch with the 20‐mer poly A linker was employed in the subsequent investigation.

### 2.2 Enhanced Inhibition Ability of the Allosteric Switch

**Figure 2:**
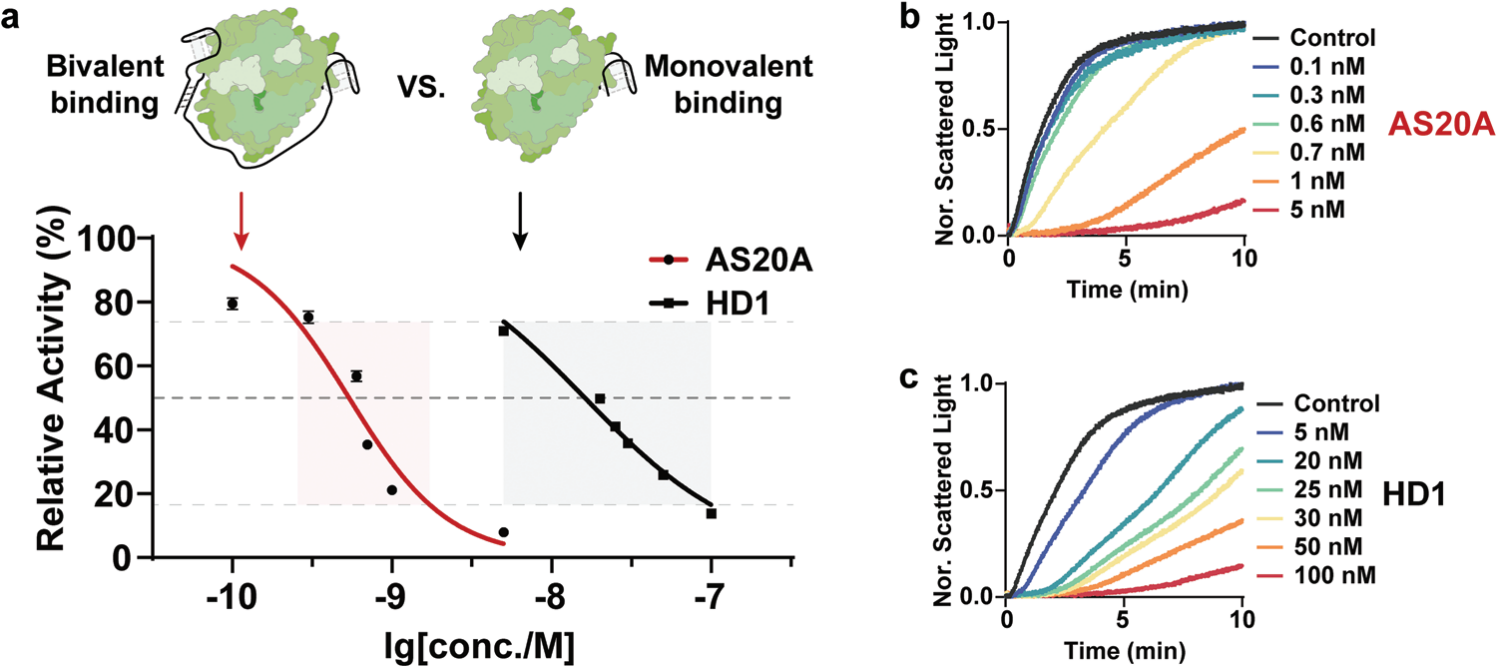
Enhanced ability of the allosteric switch to inhibit thrombin activity compared with HD1. a) Relative activity of thrombin that was preincubated with different concentrations of HD1 or AS20A (*n* = 3, mean ± SD). With the increase of AS20A from b) 0.1 to 5 nm or HD1 from c) 5 to 100 nm, the relative activity of thrombin (0.75 U mL^−1^) decreases obviously. Compared with HD1, AS20A has a lower IC50 (the corresponding allosteric effector concentration when the relative activity is 50%) and a narrower concentration window.

To accurately sense and inhibit thrombin, various thrombin binding aptamers have been screened out through the SELEX (Systematic Evolution of Ligands by Exponential Enrichment) technique,^[^ ^35^, ^36^, ^37^ ^]^ and methods such as DNA origami to spatially draw closer the two aptamers have been taken to enhance the binding and inhibition of thrombin.^[^ ^38^, ^39^, ^40^ ^]^ According to our proposed design, the synergy of HD22 will result in an increased local concentration of HD1 surrounding thrombin when the allosteric switch binds to thrombin. Furthermore, the concurrent binding (bivalent binding) of two aptamers to thrombin is bound to manifest a greater affinity toward thrombin compared with a single aptamer (monovalent binding), which exhibits a resemblance to the chelation. To validate the enhancement of bivalent binding in the inhibition of thrombin, we investigated the coagulation of fibrinogen catalyzed by thrombin that was preincubated with different concentrations of AS20A or HD1. As shown in **Figure** **2**a, nearly 30‐fold excess of HD1 is required to achieve the same inhibitory efficiency of thrombin as AS20A (IC50_[HD1]_ = 16.10 nm, IC50_[AS20A]_ = 0.5374 nm), confirming the importance of spatial proximity between two aptamers. In addition, AS20A has a narrower concentration window compared to HD1, which means that designing an allosteric switch with multiple sites provides the opportunity to engineer thrombin binding aptamer with tunable binding ability (Figure 2b,c). This agrees well with our anticipation. To certify that the enhanced anticoagulation ability is indeed due to the synergy of HD1, HD22, and poly A linker, controlled trials were conducted. Figure S3 (Supporting Information) demonstrates that neither the mixture of HD1 and HD2 (HD1+HD2) nor the 20‐mer poly A linked HD1(20A) and HD22(20A) exhibit significantly enhanced inhibition ability in comparison to HD1, let alone AS20A. Moreover, in terms of enzyme inhibition, two double‐stranded DNA with separated HD1 and HD22, HD1(20A)‐HD22(20T) and HD22(20A)‐HD1(20T), are inferior to AS20A. The results demonstrate that whether it is HD1, HD22, or the poly A linker in the allosteric switch plays an important part in enhanced inhibition of thrombin activity. Whereafter, the poly A linker as the allosteric site was chosen to execute the conversion from the *“on” state* to the *“off” state* of the allosteric switch.

### 2.3 Reversible Conversion of Conformation and Function of the Allosteric Switch

**Figure 3:**
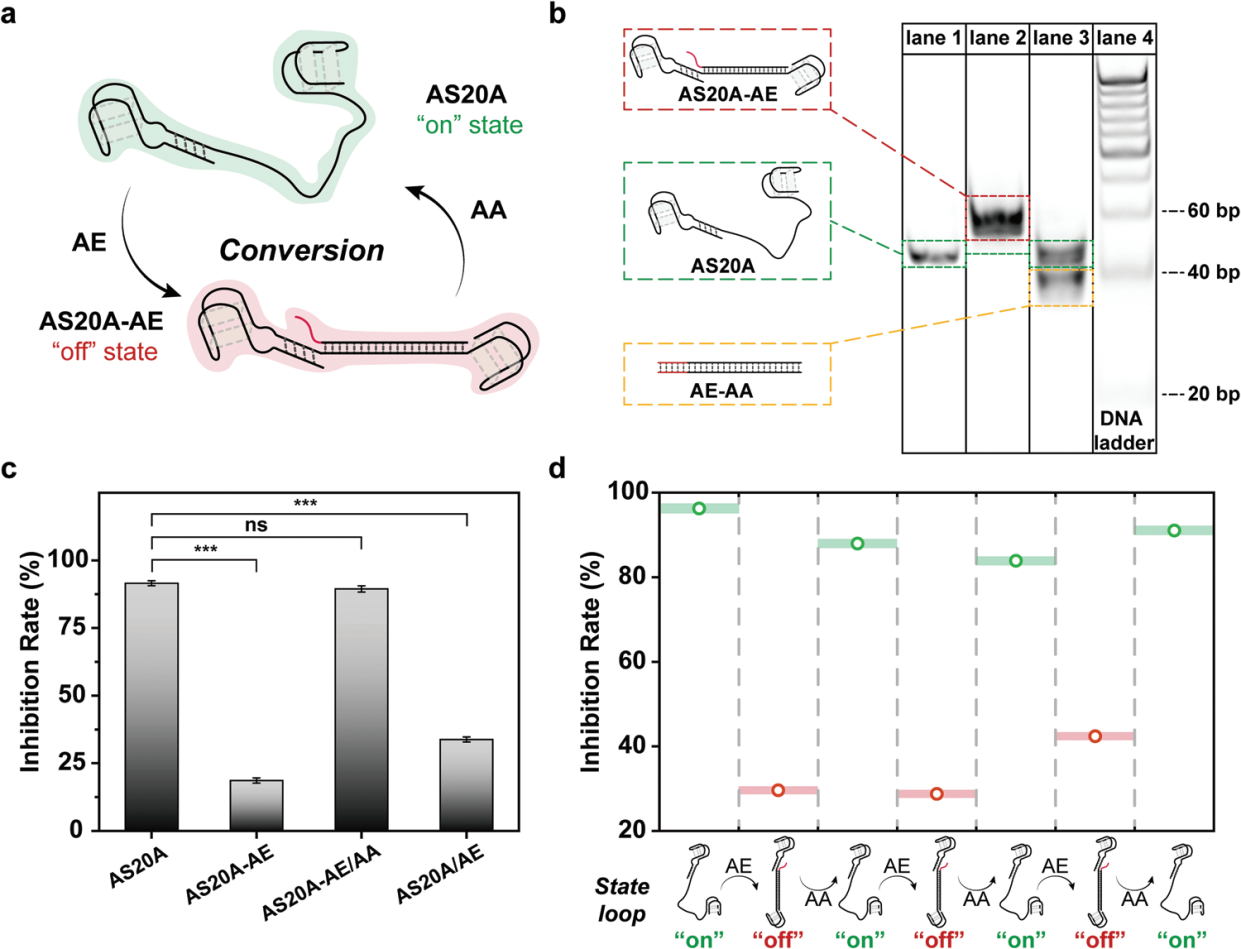
The reversible conversion of conformation and function of AS20A is realized by adding AE and AA. a) Scheme illustration of the conversion process of the allosteric switch. b) PAGE image showing the reversible state conversion of AS20A (lane 1: AS20A; lane 2: AS20A‐AE; lane 3: AS20A‐AE/AA; lane 4: 20 bp DAN ladder). c) The inhibition rate of the allosteric switches in different states to thrombin activity (*n* = 3, mean ± SD). d) The reversible inhibition of the allosteric switch (“on” state: green dots, “off” state: red dots) by alternately adding AE and AA to AS20A for three cycles (n = 3, mean ± SD). ns, *p* > 0.05; ^***^ *p* < 0.001.

To further apply this allosteric switch to selectively inhibit thrombin activity, the convertibility and the performance of switches in different states were explored. For this purpose, an allosteric effector (AE) and an allosteric antidote (AA) were introduced to perform the state conversion of the allosteric switch. Initially, the AS20A is in the *“on” state* and the poly A linker is a single strand that can fold freely, thus the appropriate conformation can be adopted by the active site for binding to thrombin and the local spatial proximity of HD1 and HD22 equips the allosteric switch in *“on” state* with stronger thrombin activity inhibition. Once AE is added, AS20A will convert into the *“off” state* with the formation of AS20A‐AE complex via complementary base pairing. Due to the formation of the rigid double helix linker, the active site is split into two spatially separate parts that are unable to bind thrombin synergistically hence the inhibition ability of AS20A is weakened. Nevertheless, after the accession of AA, the TMSD reaction drives the separation of AE from AS20A‐AE complex and the reconversion of the *“on” state* AS20A (**Figure** **3**a). The results of the polyacrylamide gel electrophoresis (PAGE) demonstrate that the allosteric switches in distinct states were well‐formed and their bidirectional conversion successfully proceeded (Figure 3b). Lane 1 of the *“on” state* AS20A manifested a band with a fast migration rate, while lane 2 of the *“off” state* AS20A, AS20A‐AE complex, showed a slower one which was attributed to its increased molecular weight. In the wake of displacement triggered by AA, a new band of AE‐AA complex debuted and the band of *“on” state* AS20A recovered in lane 3. Meanwhile, the inhibition rate of AS20A to thrombin had also been quantified according to Equation (2). From the result of Figure 3c, whether it is primary *“on” state* AS20A (AS20A group) or reconverted *“on” state* AS20A (annealed with AE and then incubated with AA, AS20A‐AE/AA group), they exhibited excellent inhibition of thrombin activity. In contrast, *“off” state* AS20A (AS20A‐AE group) did not perform well in terms of enzyme inhibition. The more exciting thing is that *“on” state* AS20A preincubated with thrombin manifested weak thrombin inhibition due to the late addition of AE (AS20A/AE group). The inhibition ability of allosteric switches with different linker lengths complementary to AE was also investigated and we found that the appropriate length of poly A linker (20A) is beneficial not only to high‐affinity binding target molecules but also to efficient conformational and functional conversion (Figure S4, Supporting Information). These results suggest that the conformation and function of the allosteric switch can be converted reversibly by adding AE and AA as the allosteric effector and allosteric antidote respectively. In addition, the inhibition of AS20A on thrombin activity can be gradually weakened or restored by adjusting the ratio of added AE or AA, which further proved that the allostery‐simulated molecule switch was successfully constructed (Figure S5, Supporting Information). Then, to probe the reversibility of the allosteric switch between the *“on” state* and *“off” state*, AE and AA were alternately added to AS20A for three cycles. As shown in Figure S6 (Supporting Information), the addition of AE makes slow migration bands arise in lanes 2, 4, and 6. The addition of AA brings the fast migration bands back in lanes 3, 5, and 7 accompanied by the accretion of AE‐AA complex band, which are coincident with the results in Figure 3b. Analogously, the inhibition rate of the allosteric switch presents a pulsed trend along with the alternate addition of AE and AA (Figure 3d). Taken together, these results confirm that the allosteric switch has satisfactory controllability on inhibition of thrombin activity. At the same time, it should be noted that the allosteric effector and the allosteric antidote are both DNA molecules, so it is possible to further realize the conversion of the allosteric switch by DNA logic circuits on demand.

### 2.4 The Allosteric Switch Plugged into the DNA Threshold Circuit for Selective Inhibition of Thrombin Activity

High levels of thrombin existence in vessels are often accompanied by the occurrence of certain cardiovascular diseases, even cancer cell migration and angiogenesis at tumor lesions.^[^ ^41^, ^42^, ^43^ ^]^ Therefore, some cancer treatment methods based on inhibiting thrombin activity are constantly being developed.^[^ ^44^, ^45^ ^]^ However, most anticoagulants are systemic administered which inevitably carries the risk of damaging the hemostasis of the patient. Therefore, developing drugs that can accurately recognize the local concentration of thrombin and selectively exert anticoagulation is highly significant. Han et al. constructed a DNA threshold circuit to achieve a specific response to high‐level thrombin.^[^ ^46^ ^]^ Recently, they have assembled the circuit into a DNA‐origami barrel for application in human plasma.^[^ ^47^ ^]^ The logical operation of DNA circuits can endow traditional functional nucleic acid with intelligence and autonomy, which brings new opportunities for its diversified application. By cascading thrombin sensing, nucleic acid information processing, and conformational regulation, we designed an integrated circuit with the allosteric switch as the bridge for selective inhibition of high‐level thrombin.

**Scheme 2:**
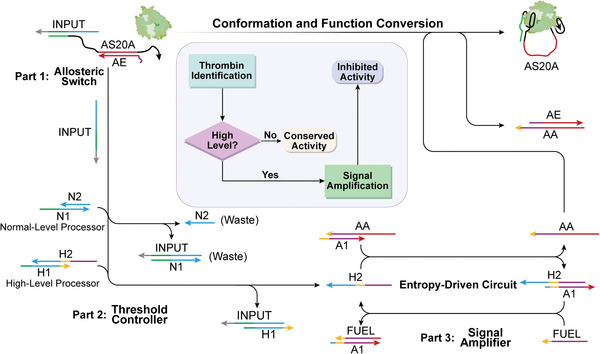
Schematic illustration of the integrated allosteric circuit in which the allosteric switch is plugged into the DNA threshold circuit for selective inhibition of thrombin activity. Part 1: The allosteric switch recognizes thrombin through aptamer (HD22) and releases INPUT strand of corresponding quantities. Part 2: The threshold controller determines whether INPUT released in the previous part is sufficient to start the entropy‐driven circuit. Part 3: INPUT passed through part 2 is converted and accessed into the signal amplifier, and then amplified into a large number of AA to execute the conformation and function conversion of the allosteric switch. Insert: The flowchart of the integrated allosteric circuit.

As shown in **Scheme** **2**, the integrated allosteric circuit consists of three parts: an allosteric switch, a threshold controller, and a signal amplifier. In the first place, the allosteric switch is responsible for transducing thrombin signals into nucleic acid signals accessed into the threshold controller and receiving instruction from the signal amplifier to implement conformation and function conversion. For this purpose, the *“off” state* allosteric switch with weak inhibition of thrombin activity was utilized as the skeleton, and a DNA strand (INPUT) partially complemented with HD22 was used as the input signal. Due to the spatial structure change of HD22 caused by thrombin binding, INPUT will be released and its quantity is positively correlated with thrombin concentration. In the second place, the threshold controller, the core of thrombin concentration calculation in the integrated circuit, is made up of a pair of dsDNA that can carry out the TMSD reaction triggered by INPUT. Compared with the high‐level processor (HLP), the normal‐level processor (NLP) possesses a longer naked toehold for strand migration, so it has a reaction priority.^[^ ^46^ ^]^ Since the small amount of INPUT released by thrombin at the normal level is entirely consumed by NLP, the subsequent circuit will not be accessed. While the INPUT released by high‐level thrombin can completely consume NLP, the rest will activate HLP and be amplified by the entropy‐driven signal amplifier into a good deal of AA as the instruction in the third place. Upon receipt of instruction, the *“off” state* allosteric switch will be converted into the *“on” state* with effective inhibition of thrombin activity. Thus, employing the integrated allosteric circuit which blends self‐sensing, threshold, and allostery, the selective and automatic inhibition of thrombin activity can be realized based on the allosteric switch in principle.

Accurate sensing of thrombin concentration is the decisive factor for the expected activity inhibition in the integrated allosteric circuit. To construct an allosteric switch for stable loading of an input signal, the binding site in AS20A was optimized. As described in Figure S7a (Supporting Information), the length of the 5′ end of AS20A was extended to avoid signal leak. The result of fluorescence intensity shows that AS20A with two extended bases in the 5′ end has preferable binding stability with INPUT (Figure S7b, Supporting Information). Then the assembly of the *“off” state* allosteric switch and INPUT was characterized by PAGE. The appearance of slower bands in lanes 3, 6, and 9 proved the successful assembly of the INPUT‐AS20A‐AA triplex (IAA), while the weakened nonspecific band in lane 9 indicated that AS20A with two extended bases bonded to INPUT more stably (Figure S7c, Supporting Information). In addition, to investigate whether the prolonged two bases and the binding of INPUT will interfere with the function of the allosteric switch, the inhibition efficiency of prolonged AS20A to thrombin was subsequently tested and the results are shown in Figure S8 (Supporting Information). The results demonstrate that the extension does not affect the controllability of conformation and function of AS20A. Perhaps because the binding between thrombin and aptamer mainly depends on the stable rings of the formed G‐quadruplex.^[^ ^48^, ^49^ ^]^ Moreover, the complement with INPUT also showed a negligible impact on the function of AS20A, which laid the foundation for integrating the allosteric switch into the DNA circuit.

**Figure 4:**
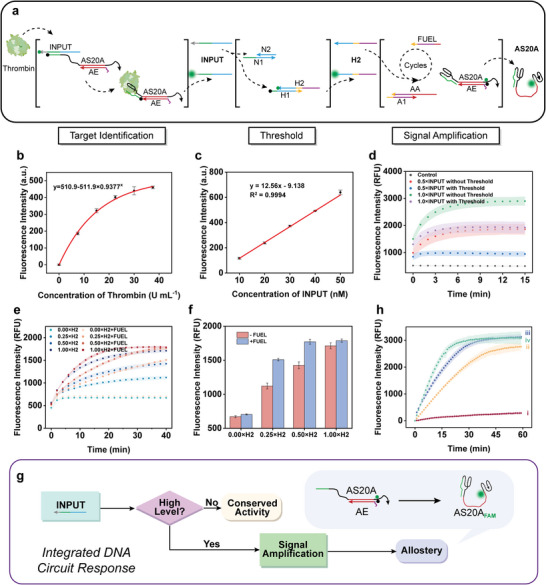
Research on the feasibility of constructing the integrated allosteric circuit step by step. a) Schematic illustration of the three‐step process of target identification, threshold, and signal amplification. b) The nonlinear fitting curve of fluorescence (*n* = 3, mean ± SD). HD22 in the IAA complex recognizes and binds thrombin to induce INPUT release and fluorescence signal recovery. c) Calibration curve of fluorescence intensity and INPUT concentration (*n* = 3, mean ± SD). d) Response of threshold controller to different intensities of INPUT (*n* = 3, mean ± SD). The entropy‐driven signal amplifier powered by FUEL enables H2 to cause e) faster and f) more significant fluorescence recovery (*n* = 3, mean ± SD). g) The flowchart of conformation and function conversion of allosteric switch initiated by INPUT through DNA threshold and signal amplifier. h) DNA circuits with and without threshold respond to normal‐level INPUT and high‐level INPUT (*n* = 3, mean ± SD): i) normal‐level INPUT + DNA circuits with threshold; ii) normal‐level INPUT + DNA circuits without threshold; iii) high‐level INPUT + DNA circuits with threshold; iv) high‐level INPUT + DNA circuits without threshold. Normal‐level INPUT: 9.383 nm; high‐level INPUT: 21.64 nm; DNA circuits with threshold: 12.5 nm NLP, 50 nm HLP, 50 nm A1‐AA, 100 nm FUEL, and 50 nm AS20A_FAM_‐AE_BHQ1_; DNA circuits without threshold: 50 nm HLP, 50 nm A1‐AA, 100 nm FUEL, and 50 nm AS20A_FAM_‐AE_BHQ1_.

Before building an entire integrated allosteric circuit, FAM‐labeled strands (INPUT, H2, and AS20A) were respectively used to investigate the performance of three important modules (**Figure** **4**a). First of all, the relationship between the concentration of thrombin and the fluorescence intensity of INPUT released in the recognition process of aptamer was determined (Figure 4b). By referencing the calibration curve of INPUT in Figure 4c, the quantities of INPUT released by thrombin addition can be determined. As a proof of concept, 3.750 U mL^−1^ thrombin (equivalent to 9.383 nm INPUT) and 11.25 U mL^−1^ thrombin (equivalent to 21.64 nm INPUT) were respectively defined as the normal level and the high level, and 12.50 nm NLP was set as the threshold of the threshold controller which is slighter higher than INPUT released by thrombin at the normal level. Then, the response of the threshold circuit to different proportions of INPUT was studied. The added INPUT (0.5× or 1.0×) was consumed preferentially by NLP so the INPUT equal to or below the threshold (0.5×) did not active the subsequent circuit, while the surplus part of the high‐intensity INPUT (1.0×) caused the release and fluorescence recovery of H2 (Figure 4d). Similarly, the performance of entropy‐driven signal amplifiers with and without FUEL as energy supply was compared employing different proportions of H2. We found that the signal amplifier powered by FUEL had a faster and more significant response (Figure 4e,f), especially for medium signal intensities (0.25× and 0.50×). Finally, as shown in Figure 4g, we investigated the performance of the threshold controller responding to signals below or above the configured threshold. The similar responses in Figure 4d,h show that the threshold circuit performs well, and the approximate maximum of fluorescence intensity indicates that the signal amplifier is vital for the conformation conversion of the allosteric switch as well. From these results, it is clear that the INPUT signal carried on IAA can be released in a thrombin concentration‐dependent manner, and the threshold controller and the signal amplifier can cascade respond to the different INPUT intensities, and perform corresponding operations.

**Figure 5:**
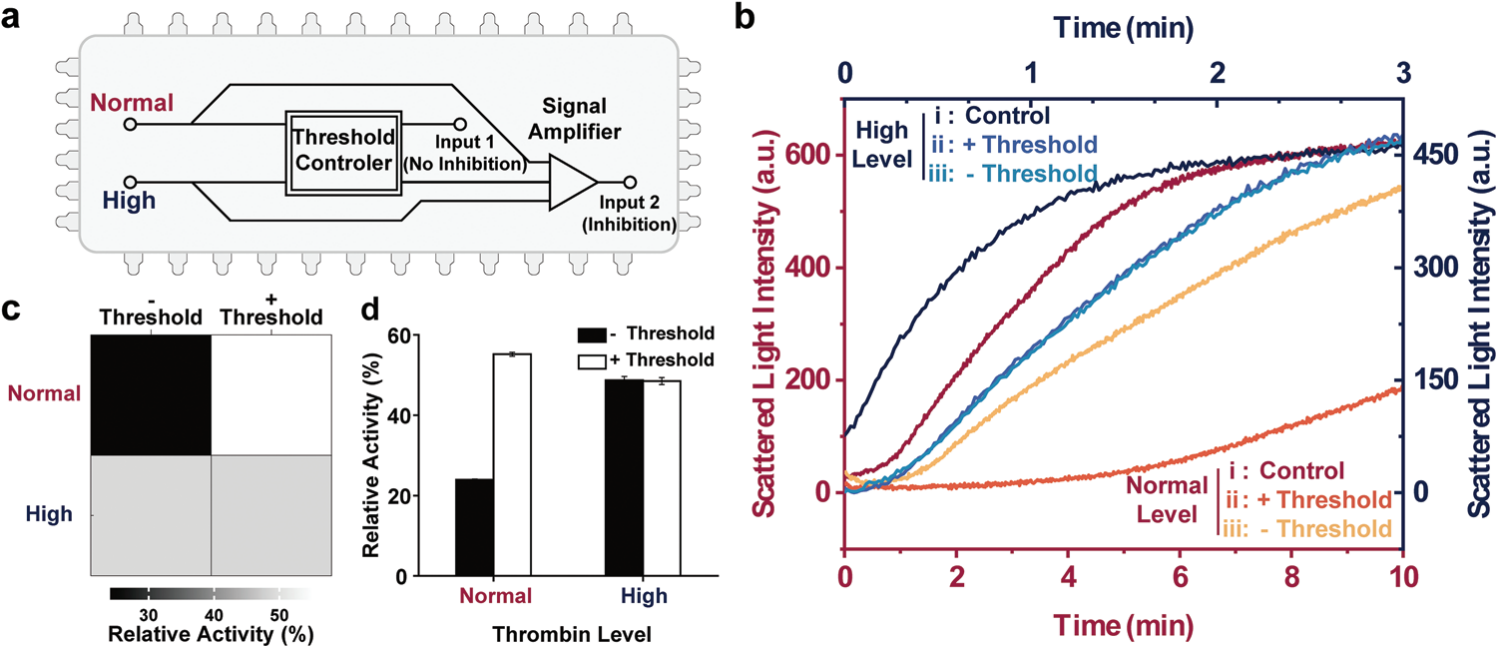
The integrated allosteric circuit differentiates the normal‐level and high‐level thrombin and inhibits thrombin activity selectively. a) Schematic illustration of the integrated allosteric circuit response to different levels of thrombin. b) Measurements of thrombin‐catalyzed cleavage of fibrinogen (1.25 mg mL^−1^) under different concentration and incubation conditions. Normal‐level thrombin: 3.75 U mL^−1^ (red series); high‐level thrombin: 11.25 U mL^−1^ (blue series). i) control: 50 nm IAA; ii) integrated allosteric circuit with threshold: 50 nm IAA, 12.5 nm NLP, 50 nm HLP, 50 nm A1‐AA, 100 nm FUEL; iii) integrated allosteric circuit without threshold: 50 nm IAA, 50 nm HLP, 50 nm A1‐AA, 100 nm FUEL. c) Heat map and d) histogram of the relative activity of thrombin at normal and high levels after incubation with two kinds of DNA circuit (*n* = 3, mean ± SD).

Given the above, two different concentrations of thrombin (normal‐level and high‐level) were used to explore the performance of the integral allosteric circuit in self‐sensing and automatic selective inhibition of enzyme activity, and a circuit without threshold setting was constructed as a comparative trial (**Figure** **5**a). As shown in Figure 5b, the activity of normal‐level thrombin (red series) incubated with the integrated allosteric circuit was inhibited slightly (red, ii line) compared to the control group incubated with only IAA (red, i line), and this slight inhibition may be attributed to the signal leakage of the DNA circuit. In contrast, the activity of high‐level thrombin (blue series) decreased significantly. However, due to the excess thrombin (three times the concentration used in the enzyme activity test) beyond the tolerance range of the allosteric switch, the activity of thrombin was still high (blue, i and ii line). This speculation was confirmed by incubating normal‐level (red, iii line) and high‐level (blue, iii line) thrombin with the DNA circuit without NLP for threshold control and the inhibition results of different ratios of AS20A on thrombin activity (Figure S9, Supporting Information). There is no obvious difference between the relative activity of high‐level thrombin incubated with the integrated allosteric circuit and that incubated with the allosteric circuit without the threshold (Figure 5c,d). While the activity of normal‐level thrombin was almost completely inhibited by the allosteric circuit without the threshold. These results demonstrate that the integrated allosteric circuit can perform the sensing and thrombin activity inhibition programs automatically. Despite the high‐level thrombin cannot be inhibited entirely, the advantage of self‐sensing and the threshold circuit over traditional regulatory methods have been shown. Overall, the integrated allosteric circuit composed of the allosteric switch, threshold circuit, and entropy‐driven signal amplifier can execute self‐sensing and concentration analysis of thrombin, thus realizing the selective inhibition of thrombin activity.

This work provides a universal paradigm for further exploration and application of biomimetic functional devices based on allostery. Compared with other artificial molecules designed based on the underlying logic of allostery,^[^ ^23^, ^24^ ^]^ the conformation and function of our allosteric switch are controlled by the instructions issued by the DNA circuit after analyzing the external environment. This regulation strategy should enable the allosteric switch as a basic module to access various molecular circuits on demand, showing great application potential. However, there are still some problems to overcome. First, the *“off” state* allosteric switch manifests an unwanted inhibition of thrombin activity, which may be further alleviated by blocking HD1. Second, redundant DNA strands are used in DNA circuits that result in low molecule utilization, thus simpler DNA circuits need to be developed to achieve the necessary operation for conformation and function conversion of the allosteric switch. Third, the inhibition efficiency of high‐level thrombin is unsatisfactory, so it is necessary to combine the allosteric switch with advanced aptamers with more outstanding thrombin inhibition, such as NU172.^[^ ^50^, ^51^ ^]^ Furthermore, as a prototype of the application of the allostery strategy, the allosteric switch can be utilized to compose multifunctional devices. For example, combined with the feedback circuit, real‐time monitoring of thrombin concentration and on‐demand control of thrombin activity contribute to stably performing the function of thrombin. In addition, coupled with the SELEX technique, the allosteric switch is available for the screening of aptamers with multiple binding sites.

## 3 Conclusion

In summary, we introduced the concept of bionics into the structure design of allosteric molecules, then based on this, developed an allosteric DNA switch with steerable inhibition ability for thrombin. Compared with HD1, the allosteric switch possesses enhanced inhibition of thrombin that is attributed to the synergistic effect of HD22. Meanwhile, by utilizing the fundamental properties of nucleic acid, the dynamic allosteric switch is further subject to the control of the DNA threshold circuit for realizing automatic concentration sensing and activity inhibition of thrombin. This integrated diagnosis and treatment method can achieve on‐demand inhibition of thrombin activity that may be able to avoid the risk of systemic bleeding caused by traditional anticoagulants. Beyond that, our study can be seen as a foray into the artificial control of biomacromolecules that include but are not limited to proteins and riboswitch. Thus, this allosteric switch equipped with self‐sensing and information processing modules puts a new slant on research of allosteric mechanisms and further application of allosteric tactics in chemical and biomedical fields.

## 4 Experimental Section

### Preparation of the Allosteric Switch and DNA Circuits

All DNA strands (100 µm) used in this study were dissolved in TAE buffer (25 mm Tris‐base, 150 mm NaCl, 5 mm KCl, 1 mm MgCl_2_, 1 mm CaCl_2_, pH 7.4) and stored at −20 °C. To form the different DNA complexes (the allosteric switches and the double‐stranded DNA required for building circuits), the specific component DNA strands were mixed in an equimolar ratio and heated to 95 °C for 5 min, then cooled slowly to room temperature then placed in a refrigerator for further use.

### Gel Electrophoresis

To investigate the assembly and state conversion of the allosteric switch, 9% native polyacrylamide gel electrophoresis was performed in 1×TBE buffer (90 mm Tris‐base, 90 mm Boric acid, 2 mm EDTA, pH 8.3). Before loading into the gel, different samples (10 µL, 1 µm) were mixed with 100×SYBR Gold (2 µL) and 6×loading buffer (2 µL). After running for 45 to 60 min at 90 V, the gel was imaged on the Molecular Imager Gel system.

### Thrombin Activity Assays

The activity of thrombin was assessed by measuring the kinetic of light scattering at 650 nm in the process of the polymerization of fibrinogen catalyzed by thrombin. Thrombin (100 and 10 U mL^−1^) was dissolved in PBS and then stored at −20 °C. Place fibrinogen (10 mg mL^−1^) freshly prepared with 0.9% NaCl solution on ice for later use. To test the catalytic reaction without allosteric switches, thrombin (9 µL, 10 U mL^−1^) and fibrinogen (18 µL, 10 mg mL^−1^) were diluted with TAE buffer, respectively, and thrombin was kept at 25 °C for 10 min, then they were mixed for activity assay. To test the inhibition of DNA strands, the thrombin was mixed with different DNA strands (15 µL) and then followed a similar process as before mentioned. To examine the performance of DNA threshold circuits, normal‐level (3.75 U mL^−1^) and high‐level (11.25 U mL^−1^) thrombin were respectively incubated with IAA(50 nm), the integrated allosteric circuit with threshold (50 nm IAA, 12.50 nm N1‐N2, 50 nm H1‐H2, 100 nm A1‐AA, and 100 nm FUEL), and the integrated allosteric circuit without threshold (50 nm IAA, 50 nm H1‐H2, 100 nm A1‐AA, and 100 nm FUEL) at 25 °C for 30 min before the scattered light intensity test.

### State Conversion of Allosteric Switch

The state conversion was verified by the native PAGE. To realize the state conversion of the allosteric switch, AS20A (1 µL, 12 µm) was annealed, then AE (12 µm) and AA (12 µm) were added alternately with 1.1 times the volume of the former and the mixture was incubated at 25 °C for 30 min each time. Finally, the mixtures were diluted to 12 µL by TAE buffer.

### Fluorescence Intensity and Kinetics Assays

The fluorescence intensity assay was finished with an F‐7100 spectrophotometer (excitation: 494 nm, emission: 528 nm). To determine the fluorescence quenching effect, annealed DNA complexes (50 nm) were tested. To quantify the released INPUT strand, different volumes of thrombin (100 U mL^−1^) were mixed with annealed allosteric switch (INPUT_FAM_/AS20A_BHQ1_/AE, 30 µL, 200 nm), then the mixture was diluted by TAE buffer to 120 µL and tested after incubation at 25 °C for 10 min. To fit the calibration curve of INPUT, the fluorescence intensity of INPUT_FAM_ with different concentrations were tested. The fluorescence kinetics assay was conducted with a multifunctional microplate reader to study the response DNA threshold circuit. Threshold: 25 nm or 50 nm INPUT, 25 nm N1‐N2, 50 nm H1_BHQ1_‐H2_FAM_. Signal amplification: H2 (12.5 nm, 25 nm, or 50 nm), 100 nm A1‐AA, 100 nm FUEL, 50 nm AS20A_FAM_‐AE_BHQ1_. Compared with the circuit with the threshold (12.50 nm N1‐N2, 50 nm H1‐H2, 100 nm A1‐AA, 50 nm AS20A_FAM_‐AE_BHQ1_, and 100 nm FUEL), the circuit without the threshold was lack in N1‐N2 (50 nm H1‐H2, 100 nm A1‐AA, 50 nm AS20A_FAM_‐AE_BHQ1_, and 100 nm FUEL). Then INPUT (9.383 and 21.64 nm) was added to the above two groups and the fluorescence kinetics assay was performed immediately.

### Statistical Analysis

Raw scattered light signals were pre‐processed by subtracting the minimum in the group, and further normalization facilitates the comparison of data with regular changes in the same group. The relative activity was defined as the ratio of the initial rate of catalytic reaction between the experimental group and that of the control group, and the inhibition rate is defined as the ratio of the reduction between the two rates to the initial reaction rate of the control group (Equations (1) and (2)). In the processing of fluorescence kinetics and fluorescence intensity data, the fluorescence signal of the blank group (that has the buffer solution only) was deducted. All the data were presented in the form of mean ± SD, and three independent experiments were conducted for each statistical analysis. The comparison of two groups were carried out using the two‐sided Student's *t*‐test (ns, *p* > 0.05; ***p < 0.001). Microsoft Excel 2021, OriginPro 2021, and GraphPad Prism 8.2.1 were used for statistical analysis.

## Conflict of Interest

The authors declare no conflict of interest.

## Supporting information

Supporting Information

## Data Availability

The data that support the findings of this study are available from the corresponding author upon reasonable request.
