# Supplementary material for: Aptamer‐Based DNA Allosteric Switch for Regulation of Protein Activity
Source: Adv Sci (Weinh). 2024 Jun 12;11(30):2402531. doi: 10.1002/advs.202402531 (PMC11321679; doi:10.1002/advs.202402531)
Supplement: Supplementary file 1 — Supporting Information [file ADVS-11-2402531-s001.docx]

Supporting Information

Aptamer-based DNA Allosteric Switch for Regulation of Protein Activity

Hongzhi Sun, Di Zhao, Yating He, Hong-Min Meng,* and Zhaohui Li*

**1. Experimental Section**

*Reagents*: All DNA sequences used in this work were purified by HPLC and synthesized by Sangon Biotech Co., Ltd. (Shanghai, China) and their detailed sequences were listed in **Table S1**. Human α-thrombin and fibrinogen were purchased from Sigma-Aldrich (St. Louis, MO, USA). Acrylamide/Bis solution (30% (w/v)) were obtained from Sangon Biotech Co., Ltd. (Shanghai, China). SYBR Gold were supplied by Thermo Scientific HyClone (Waltham, MA, USA). PBS were obtained from Wuhan Servicebio Technology Co., Ltd. (Wuhan, China). 20 bp DNA ladder was brought from Takara (Tokyo, Japan).

*Apparatus*: Fluorescence and scattering light measurements were performed on a F-7100 spectrophotometer (Hitachi, Japan). Fluorescence intensity of FAM was recorded in 528 nm with an excitation wavelength of 494 nm. Scattered light intensity of fibrin was recorded in 650 nm with an excitation wavelength of 650 nm. Native polyacrylamide gel electrophoresis was imaged on a ChemiDoc XRS system (Bio-Rad, USA). Fluorescence kinetics assay was conducted with a SPARK multifunctional microplate reader (TECAN, Switzerland). Ultrapure water (≥18 MΩ) was produced using a water purification system (MilliQ, Germany).

**Table S1.** Sequences of oligonucleotides used in this work.

| **Name** | **Sequence 5’-3’** |
| --- | --- |
| HD1 | GGTTGGTGTGGTTGG |
| HD22 | AGTCCGTGGTAGGGCAGGTTGGGGTGACT |
| AS10A | AGTCCGTGGTAGGGCAGGTTGGGGTGACT**A_10_**GGTTGGTGTGGTTGG |
| AS20A | AGTCCGTGGTAGGGCAGGTTGGGGTGACT**A_20_**GGTTGGTGTGGTTGG |
| AS20A_BHQ1_ | **/BHQ1/**AGTCCGTGGTAGGGCAGGTTGGGGTGACT**A_20_**GGTTGGTGTGGTTGG |
| **Name** | **Sequence 5’-3’** |
| AS20A_FAM_ | AGTCCGTGGTAGGGCAGGTTGGGGTGACT**A_20_/FAM/**GGTTGGTGTGGTTGG |
| AS30A | AGTCCGTGGTAGGGCAGGTTGGGGTGACT**A_30_**GGTTGGTGTGGTTGG |
| AE(10A) | TGAGATGTTTTTTTTTT |
| AE(10A) | TGAGATGTTTTTTTTTTTTTTTTTTTT |
| AE(10A) | TGAGATGTTTTTTTTTTTTTTTTTTTTTTTTTTTTTT |
| AE_BHQ1_ | **/BHQ1/**TGAGATGTTTTTTTTTTTTTTTTTTTT |
| AA | AAAAAAAAAAAAAAAAAAAACATCTCAAAACAAAACCTCA |
| HD1(20A) | AAAAAAAAAAAAAAAAAAAAGGTTGGTGTGGTTGG |
| HD1(20T) | GGTTGGTGTGGTTGGTTTTTTTTTTTTTTTTTTTT |
| HD22(20A) | AGTCCGTGGTAGGGCAGGTTGGGGTGACTAAAAAAAAAAAAAAAAAAAA |
| HD22(20T) | TTTTTTTTTTTTTTTTTTTTTCAGTGGGGTTGGACGGGATGGTGCCTGA |
| AS20AL1 | TAGTCCGTGGTAGGGCAGGTTGGGGTGACT**A_20_**GGTTGGTGTGGTTGG |
| AS20AL1_BHQ1_ | **/BHQ1/**TAGTCCGTGGTAGGGCAGGTTGGGGTGACT**A_20_**GGTTGGTGTGGTTGG |
| AS20AL2 | TTAGTCCGTGGTAGGGCAGGTTGGGGTGACT**A_20_**GGTTGGTGTGGTTGG |
| AS20AL2_BHQ1_ | **/BHQ1/**TTTAGTCCGTGGTAGGGCAGGTTGGGGTGACT**A_20_**GGTTGGTGTGGTTGG |
| INPUT | CAAGAATAATACACAACCACGGACTAAA |
| INPUT_FAM_ | CAAGAATAATACACAACCACGGACTAAA**/FAM/** |
| N1 | AGTCCGTGGTTGTGTATTATTCTTG |
| N2 | CAAGAATAATACACA |
| H1 | GTGGTTGTGTATTATTCTTGAGATG |
| H2 | CAAAACAAAACCTCATCTCAAGAATAATACACA |
| H1_BHQ1_ | **/BHQ1/**GTGGTTGTGTATTATTCTTGAGATG |
| H2_FAM_ | CAAAACAAAACCTCATCTCAAGAATAATACACA**/FAM/** |
| A1 | TGAGATGAGGTTTTGTTTTGAGATG |
| FUEL | CATCTCAAAACAAAACCTCA |

**2. Results and Discussion:**


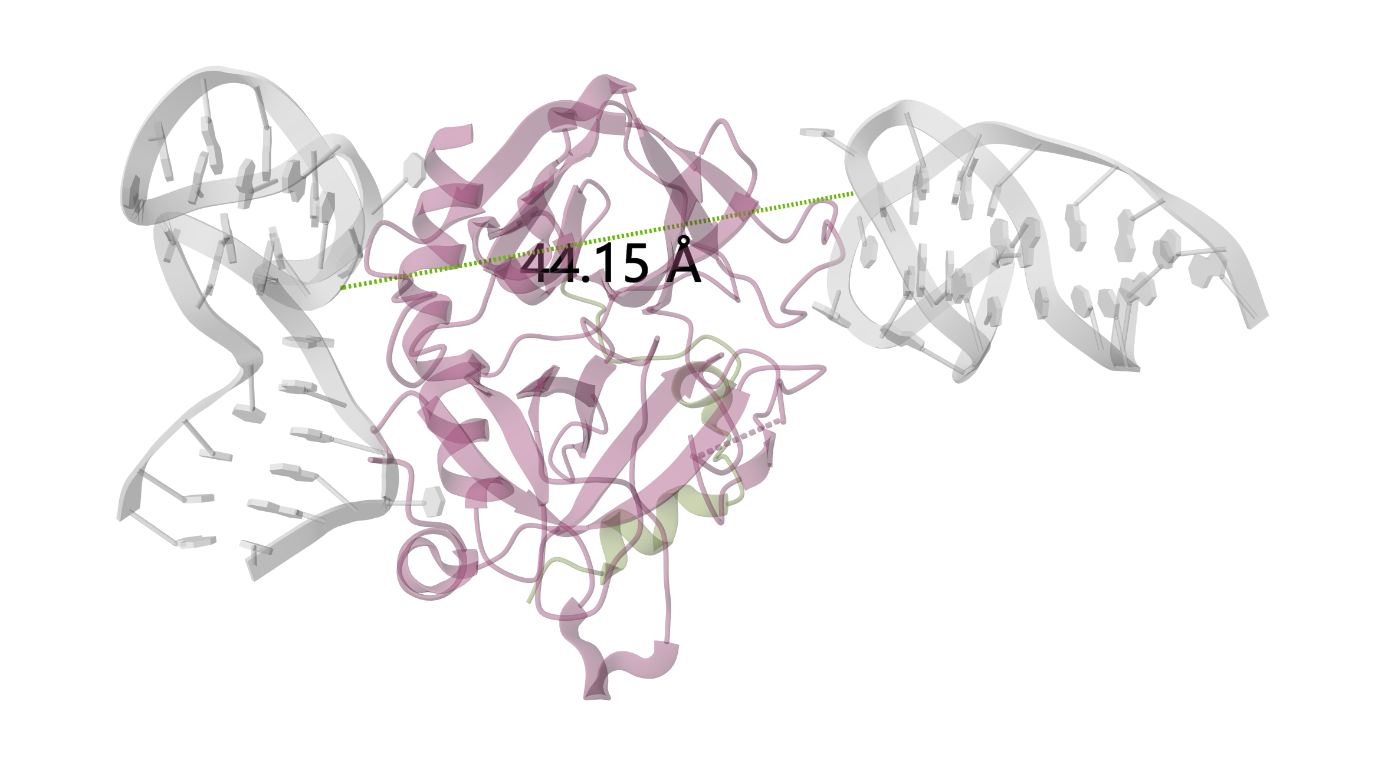


**Figure S1.** The distance between two binding sites in the NU172-Thrombin-HD22_27mer sandwich structure (PDB DOI: https://doi.org/10.2210/pdb7NTU/pdb).

**
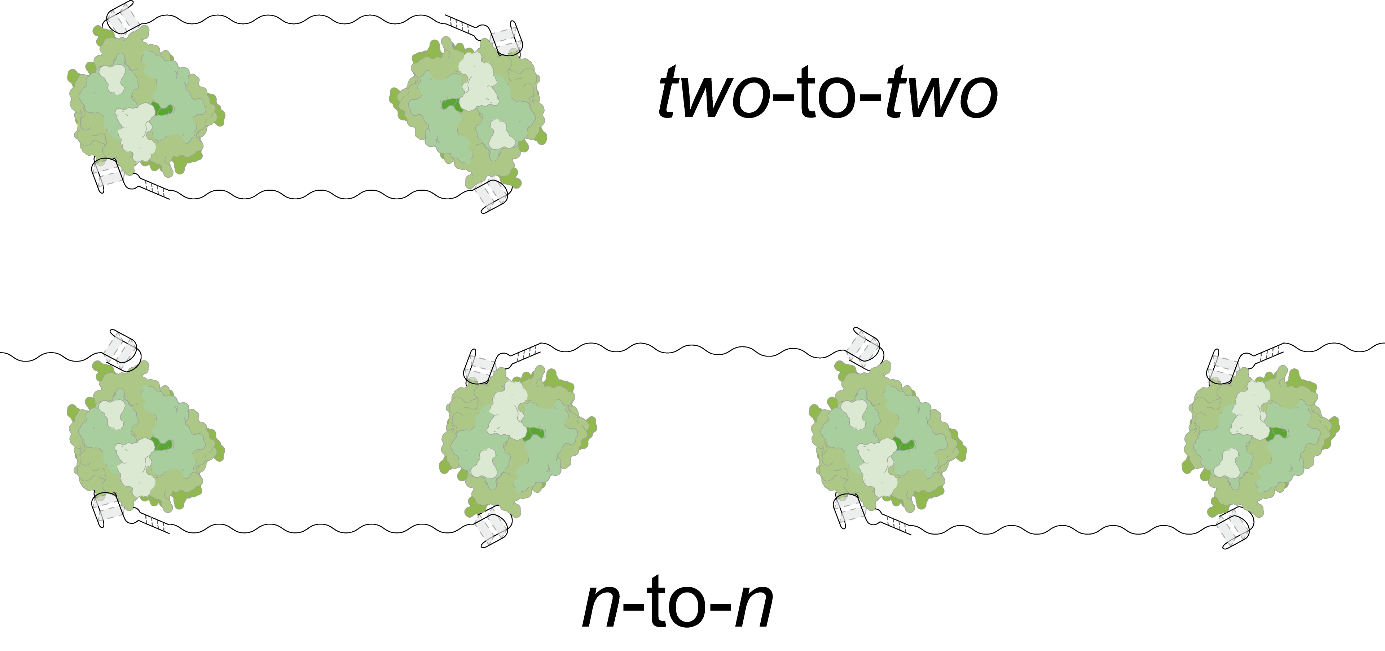
**

**Figure S2.** Possible binding modes between the allosteric switch and thrombin when DNA linker is too long.

**
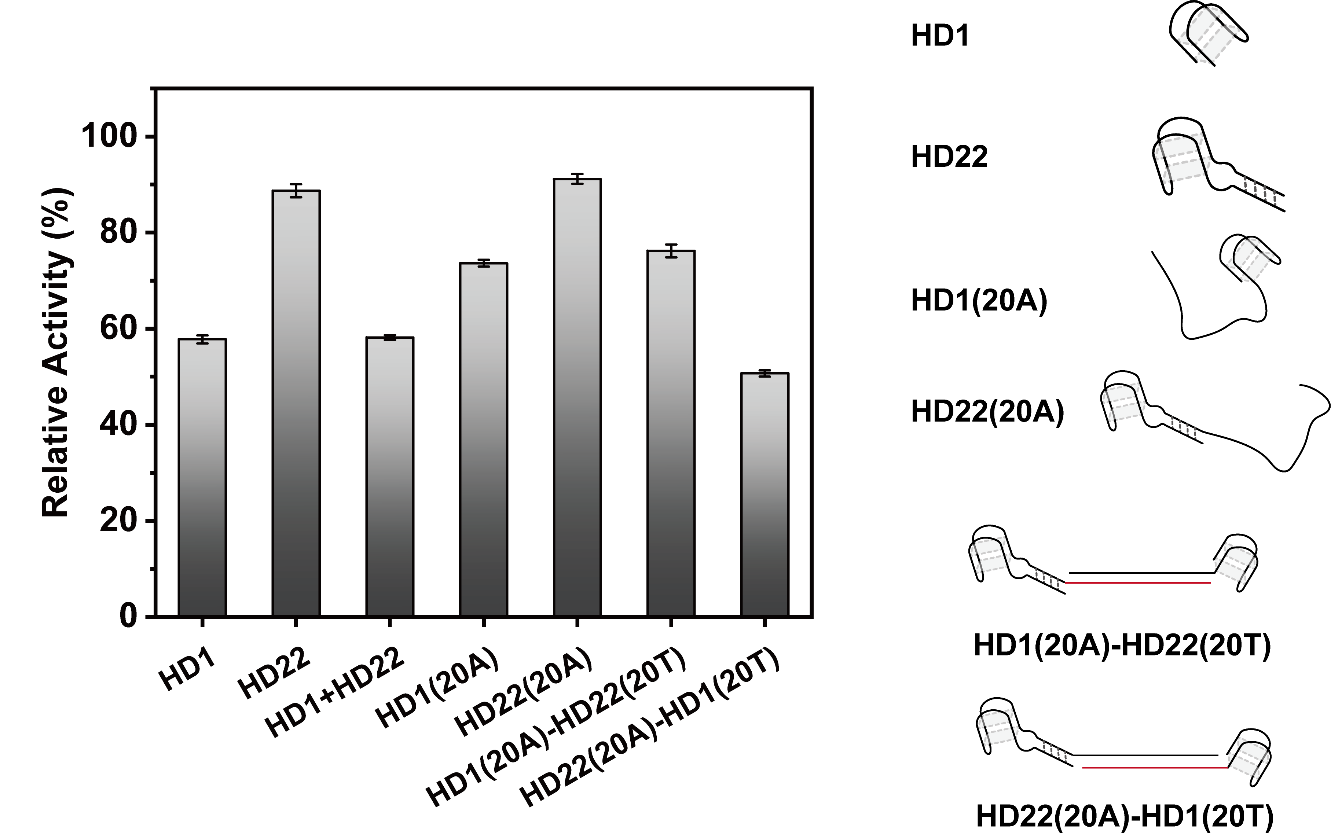
**

**Figure S3.** The relative activity of thrombin incubated with different aptamers and linker-connected aptamers (n=3, mean ± SD).

**
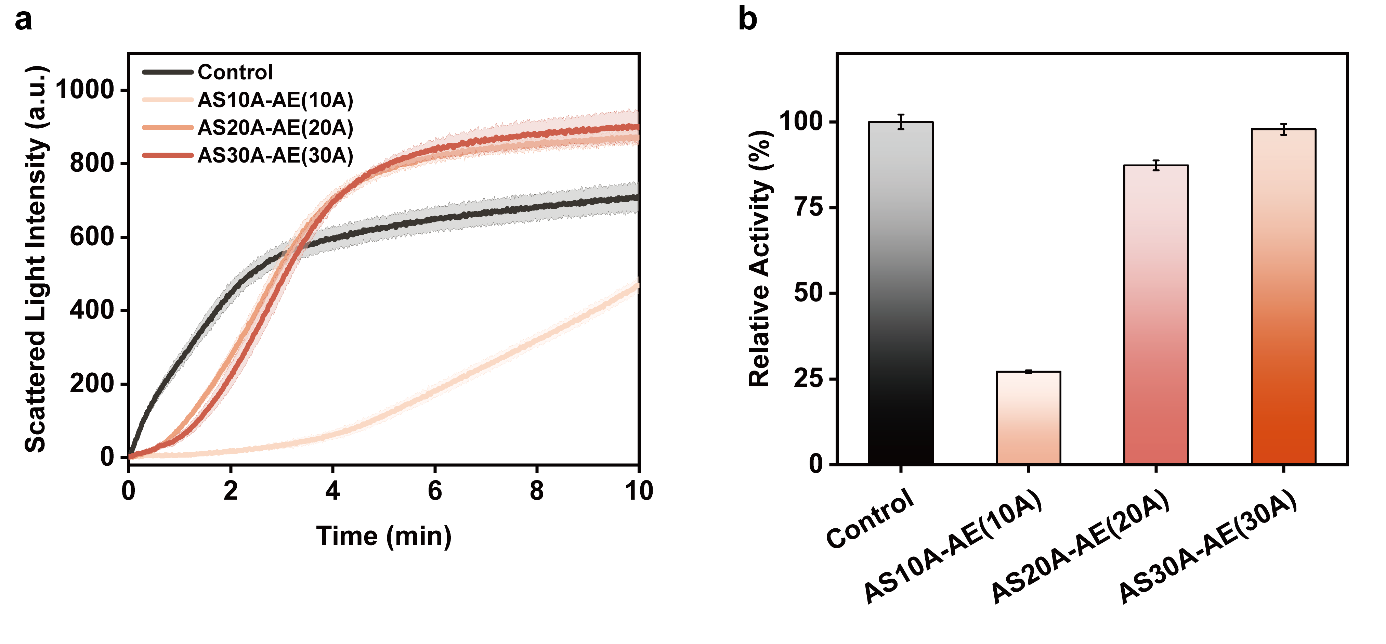
Figure S4.** Investigation of the inhibition ability of allosteric switches with different linker lengths complementary to AE. Three kinds of AE corresponding to poly A linker of the allosteric switch were adopted. (a) The kinetics of light scattering due to the conversion of fibrinogen into fibrin catalyzed by thrombin (Control) or thrombin that were preincubated with different allosteric switches (ASnA-AE(nA), n=10, 20, and 30). The fill area under the solid lines is the error bar (n=3, mean ± SD). (b) The relative activity of thrombin that was preincubated with different allosteric switches (n=3, mean ± SD).

**
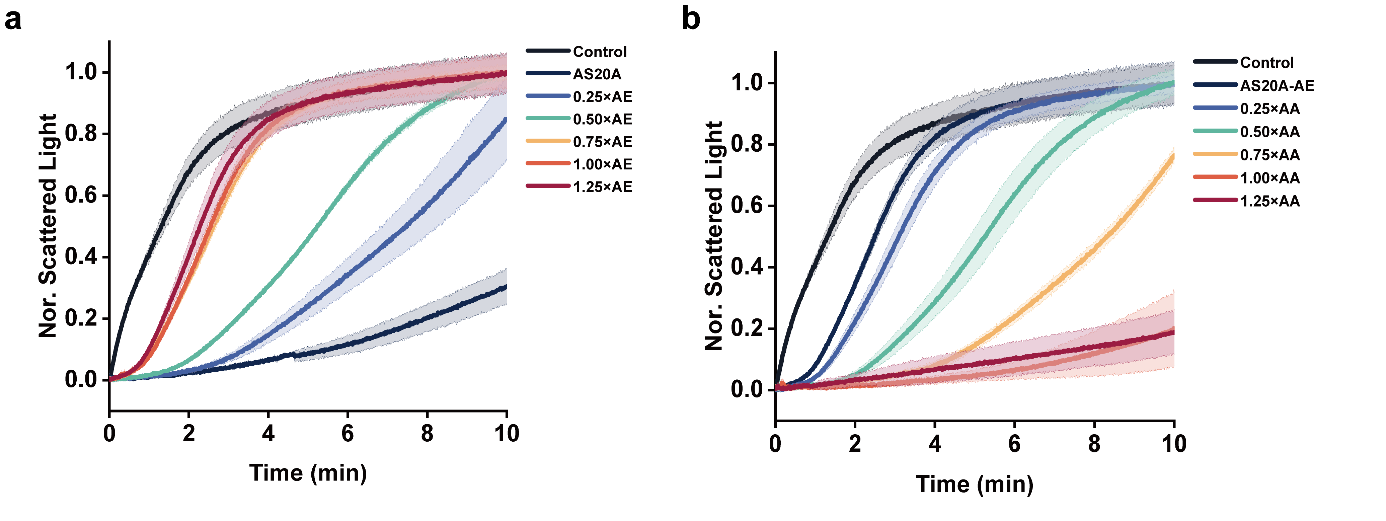
**

**Figure S5.** The reversible regulation of AS20A on the inhibition of thrombin activity. The addition of AE (a) mitigated the inhibition of AS20A on thrombin activity, while the addition of AA (b) could eliminate the impact of AE on AS20A (n=3, mean ± SD).

**
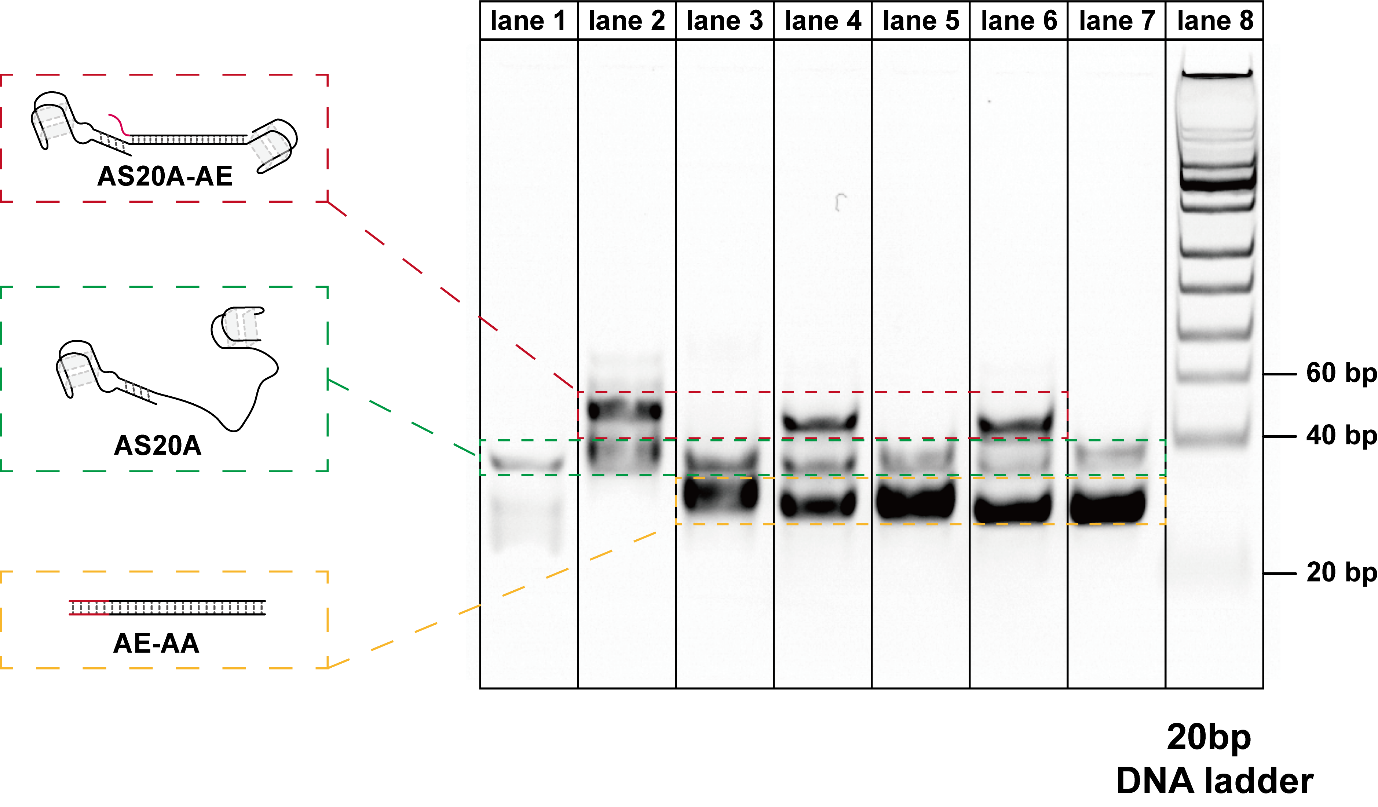
**

**Figure S6.** PAGE image of reversible conversion of AS20A by alternately adding AE and AA to AS20A for three cycles.

**
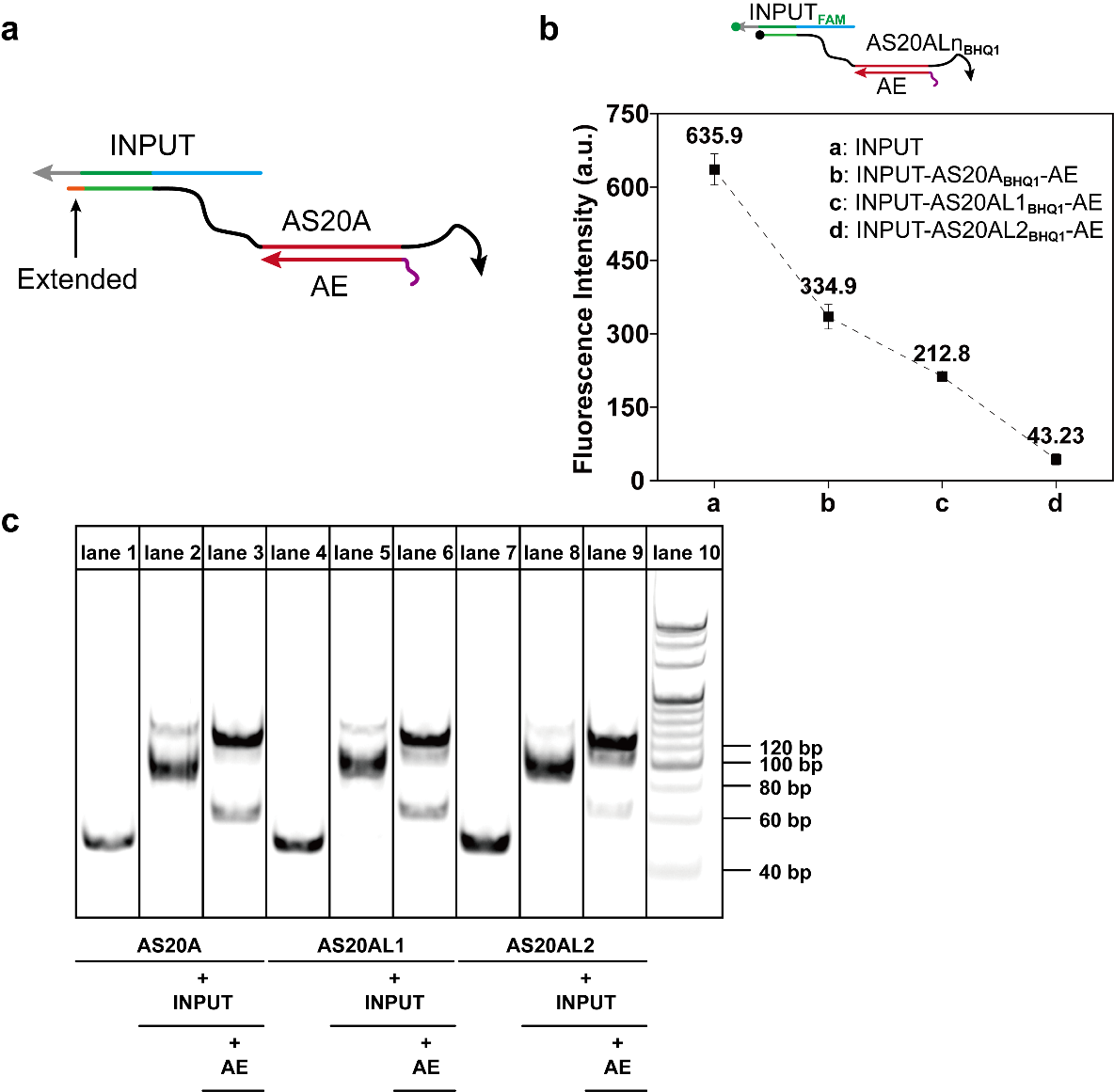
**

**Figure S7.** Study on the stability of AS20A combined with INPUT and AE. AS20A was extended in the binding site and then denoted as AS20ALn. (a) Schematic illustration of the 5’ end of AS20A extended for avoiding INPUT leak. (b) Fluorescence intensity of INPUT_FAM_ and INPUT_FAM_-AS20ALn_BHQ_-AE triplex (n=3, mean ± SD). (c) PAGE image of AS20ALn combined with INPUT and AE (lane 1: AS20A, lane 2: AS20A-INPUT, lane 3: AS20A-INPUT-AE, lane 4: AS20AL1, lane 5: AS20AL1-INPUT, lane 6: AS20AL1-INPUT/AE, lane 7: AS20AL2, lane 8: AS20AL2-INPUT, lane 9: AS20AL2-INPUT-AE, lane 10: 20bp DAN ladder).

**
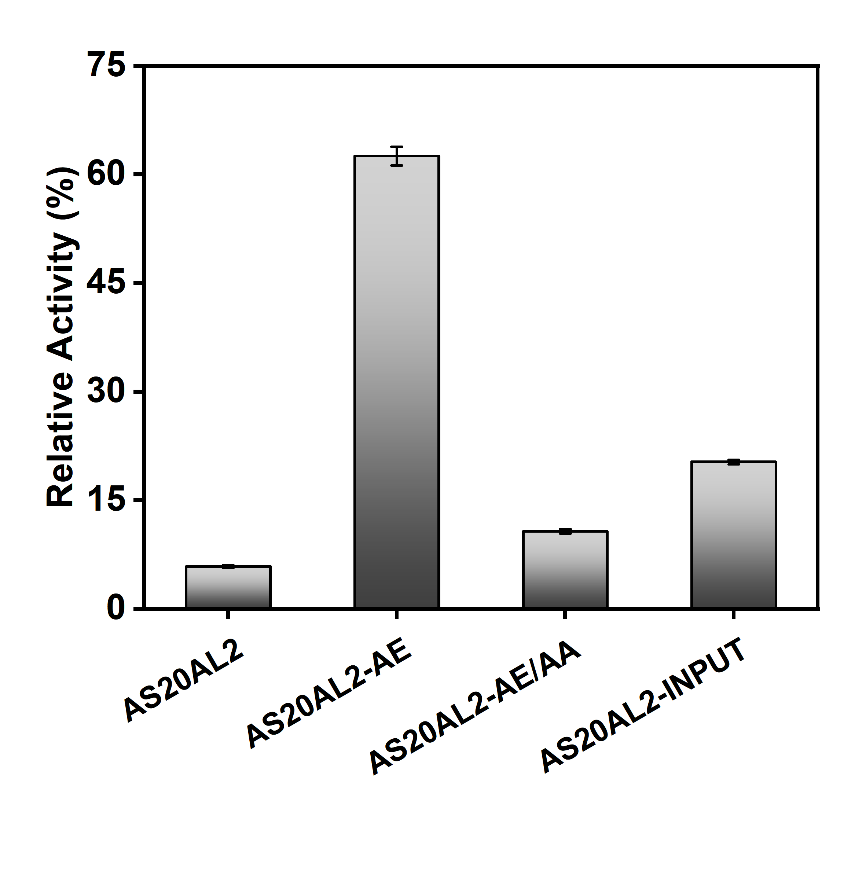
**

**Figure S8.** The relative activity of thrombin that was preincubated with AS20AL2 in different states (n=3, mean ± SD).

**
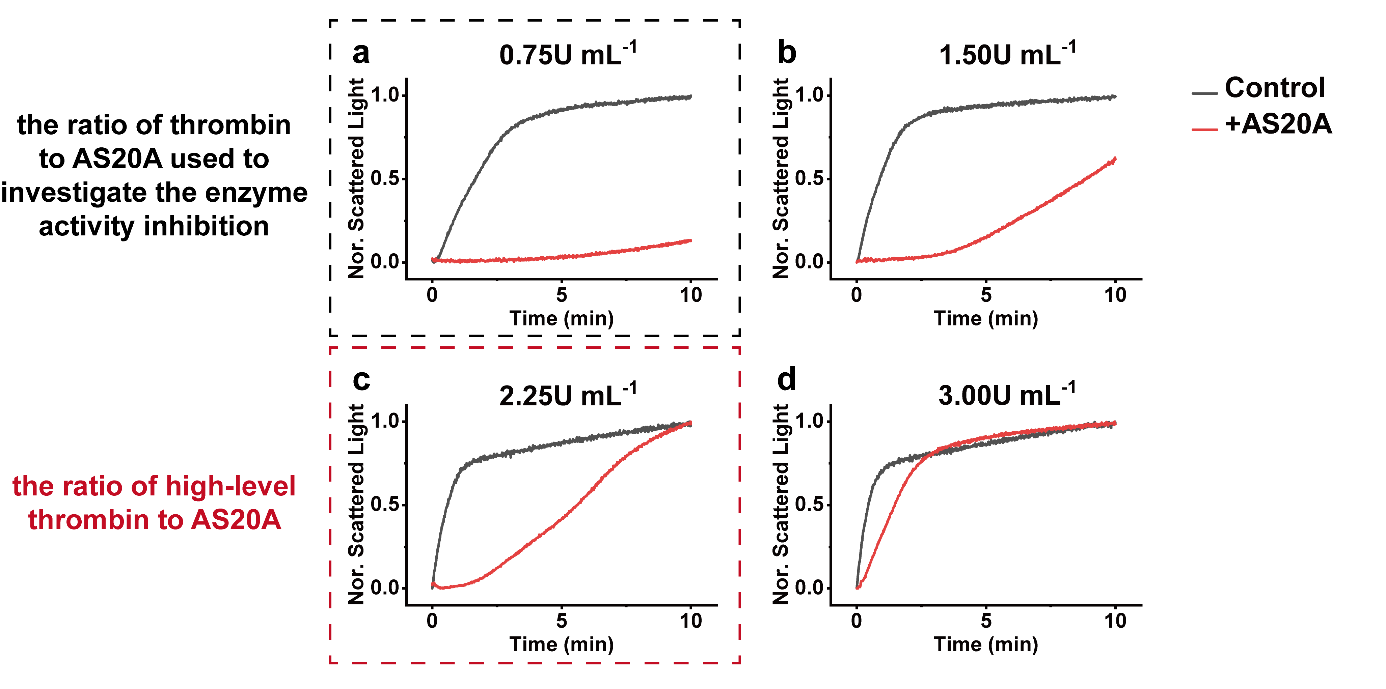
**

**Figure S9**. Normalized scattered light enhancement due to fibrinogen cleavage caused by thrombin and thrombin incubated with 10 nM AS20A. Thrombin concentration: (a) 0.75 U mL^-1^, equal proportion to normal-level, (b) 1.50 U mL^-1^, (c) 2.25 U mL^-1^, equal proportion to high-level, (d) 3.00 U mL^-1^.
